# Atherosclerosis by Virus Infection—A Short Review

**DOI:** 10.3390/biomedicines10102634

**Published:** 2022-10-19

**Authors:** Seang-Hwan Jung, Kyung-Tae Lee

**Affiliations:** 1 Department of Biomedical and Pharmaceutical Sciences, College of Pharmacy, Kyung Hee University, Seoul 02247, Korea; 2 Department of Pharmaceutical Biochemistry, College of Pharmacy, Kyung Hee University, Seoul 02247, Korea

**Keywords:** atherosclerosis, influenza viruses, coronavirus disease 2019 (COVID-19), severe acute respiratory syndrome (SARS) coronavirus-2 (CoV-2), hepatitis viruses, herpes simplex virus (HSV), human papillomavirus (HPV), human cytomegalovirus (HCMV), human immunodeficiency virus (HIV)

## Abstract

Atherosclerosis manifests by the thickening of artery walls and their narrowed channels through the accumulation of plaque. It is one of the most important indicators of cardiovascular disease. It can be caused by various factors, such as smoking, a high cholesterol diet, hypertension, hyperglycemia, and genetic factors. However, atherosclerosis can also develop due to infection. It has been reported that some bacteria and viruses can cause the development of atherosclerosis. Examples of these viruses are influenza viruses, herpes viruses, hepatitis viruses, or papillomaviruses, which are all prevalent and eminent globally for infecting the population worldwide. Moreover, many patients with coronavirus disease 2019 (COVID-19) showed symptoms of cardiovascular disease. In this review paper, the viruses linked to the development of atherosclerosis are introduced, and their viral characteristics, the mechanisms of the development of atherosclerosis, and the current vaccines and antiviral treatment methods are summarized.

## 1. Introduction

Atherosclerosis is a chronic disease whose prominent features are the thickening of artery walls and their narrowed channels through the accumulation of plaque, which mostly consists of fats, cholesterols, mineral crystals, and cellular waste products [1,2]. It obstructs blood flow to heart and other organs causing coronary heart disease, ischemic stroke, or peripheral vascular diseases [3]. It has been well studied that atherosclerosis can develop due to various factors, such as smoking, a high cholesterol diet, hypertension, hyperglycemia, or genetic factors [4]. Recently, it has also been suggested that inflammation and infection can trigger the development of atherosclerosis by themselves, in the absence of other risk factors [5,6]. It has been reported that some bacteria, such as *Chlamydia pneumoniae* and *Helicobacter pylori*, or various viral agents including influenza viruses, hepatitis viruses, herpes simplex viruses (HSV), human papillomavirus (HPV), human cytomegalovirus (HCMV), and human immunodeficiency virus (HIV) can provoke the development of atherosclerosis, where the feasible mechanisms are the overexpression of various cytokines, chemoattractant molecules, adhesion molecules, and growth factors after the occurrence of infections, increased oxidation and uptake of low-density lipoprotein (LDL), and increased resistance against apoptosis [7]. Moreover, according to the Centers for Disease Control, patients suffering from coronavirus disease 2019 (COVID-19) caused by the novel severe acute respiratory syndrome (SARS) coronavirus-2 (CoV-2) showed that cardiovascular disease is one of the most common comorbidity conditions, which strongly suggests cardiovascular and atherosclerosis manifestations of SARS CoV-2 [8,9]. These viruses are all prevalent and eminent globally for infecting the population worldwide and knowledge of the specific viruses provoking the development of atherosclerosis is prominent in this pandemic era. This paper is a concise review of the viral agents which have been reportedly linked to the development of atherosclerosis. This paper covers the viral characteristics, the mechanisms of atherosclerosis development, and current vaccines and antivirals altogether, to provide accurate information in a brief review format for students, scientists, and healthcare professionals who want to rapidly learn about the viruses linked to atherosclerosis. The general molecular mechanisms and pathways that are considered to be linked to the pathogenesis of atherosclerosis via the viral infection are appended in the discussion chapter.

## 2. Influenza Viruses

Influenza viruses, which infect the respiratory system, generally cause fever, a sore throat, muscle pain, coughing, fatigue, and a runny nose, but they can cause severe symptoms, such as acute lung injury, pulmonary oedema, hypoxemia, acute respiratory distress syndrome, and even cardiovascular collapse with thrombosis and acute myocardial infection [10,11,12]. They cause life-threatening systemic inflammatory syndromes by elevating adhesion molecules, chemokines, inflammatory mediators, and cytokines, such as tumor necrosis factor (TNF), interleukin-1 beta (IL-1β), and interleukin-6 (IL-6), and by hyperactivating and proliferating immune cells [13,14]. Neutrophils secrete neutrophil extracellular traps (NETs), which show cytotoxic effects on the lung endothelial cells and eventually damage the organs [15]. Moreover, the viruses can induce apoptosis of epithelial cells and remodel the structure of the endothelium to cause endothelial permeability and vascular leak via hyperactivation of cytokines and chemokines [16,17]. It has been shown at the diagnostic and practical level that influenza infection and acute myocardial infarction are highly cross-linked, and it is reported atherosclerosis can be caused by a thrombogenic environment through the platelet activation and endothelial dysfunction generated by influenza infections [18,19]. In addition, neuraminidase, a group of enzymes that cleave sialic acid during viral exit from the host cell, can induce desialylation of lipoproteins, increase the uptake of LDL, and thus enhance atherosclerosis development by increasing blood clots [20,21]. Infections by specific strains of influenza viruses can be prevented by vaccination and treated by antiviral drugs, such as oseltamivir phosphate (Tamiflu^®^), and it has been reported that successful influenza vaccination can prevent the development of cardiac diseases associated with influenza viruses [22,23].

## 3. Severe Acute Respiratory Syndrome Coronavirus-2 (SARS CoV-2)

SARS CoV-2 is a single-stranded enveloped RNA virus covered with glycoprotein spikes causing the virus to have a crown-like shape from which its Latin name corōna, meaning a crown, derives, and it belongs to the family Coronaviridae where seven human pathogenic coronaviruses have been recognized and documented: HCoV-229E, HCoV-NL63, HCoV-OC43, HCoV-HKU1, MERS-CoV, SARS-CoV, and SARS-CoV-2, among which MERS-CoV, SARS-CoV, and SARS-CoV-2 have been classified as the pandemic strains [24,25]. A global pandemic outbreak of COVID-19 is caused by the new type of coronavirus, SARS CoV-2, discovered in Wuhan in China in December 2019, whose viral structure and pathogenicity are similar to the SARS coronavirus discovered in Foshan, China in 2002 and Middle East respiratory syndrome (MERS) coronavirus discovered in 2012 [25,26]. Similar to other coronaviruses, SARS-CoV-2 must attach and penetrate host cells via endocytosis to replicate its virions inside the host, where it releases its viral genome, translates its RNA sequences to make the viral proteins, replicates its RNA genome, and assembles whole viral particles to mature and complete its replication [24]. SARS-CoV-2 targets angiotensin-converting enzyme (ACE) 2 receptors on the surface of epithelial cells of the trachea, bronchi, bronchial serous glands, and the alveoli of the human respiratory tract [25]. The increased expression of ACE2 receptors can facilitate and stimulate SARS-CoV-2 and induce more severe symptoms [26].

SARS-CoV-2 infection stimulates secretions of IL-1β, interferon-γ (IFN-γ), IFN-γ-induced protein 10kDa (IP-10), monocytic chemoattractant protein-1 (MCP-1), interleukin-4 (IL-4), IL-6, and interleukin-10 (IL-10), some of which can trigger a cytokine storm and atherosclerosis in a host [9,27]. In addition, SARS-CoV-2 acts as a complement activator in a host so that C3 and C5 are activated, and ultimately induce acute respiratory distress syndrome (ARDS) in a host [28]. Moreover, it has been reported that many patients with a SARS-CoV-2 infection had a ST-segment-elevation on electrocardiography, myocardial infarction, and acute myocarditis [29,30]. It is currently understood that COVID-19 may cause atherosclerosis due to the extremely high levels of proinflammatory cytokine produced after SARS-CoV-2 infection [8,9,27]. While few vaccines are available, some antivirals, such as Remdesivir, which is a nucleotide analogue prodrug, Molnupiravir, which is a ribonucleotide prodrug of beta-D-N4-hydroxycytidine (NHC), and Paxlovid, which is Ritonavir-Boosted Nirmatrelvir, showed activities against SARS-CoV-2 [31,32]. However, the Food and Drug Administration (FDA) has approved Remdesivir only for the treatment of COVID-19, while Molnupiravir and Paxlovid are not authorized for use in patients who are hospitalized with severe symptoms of COVID-19, but instead those two antivirals have received emergency-use authorization from the FDA for the treatment of mild to moderate symptoms of COVID-19, as the use of the antibody treatments reduced the risk of hospitalization and death [33].

## 4. Hepatitis Viruses

The hepatitis-causing viruses include Hepatitis A Virus (HAV), Hepatitis B Virus (HBV), Hepatitis C Virus (HCV), Hepatitis D Virus (HDV), and Hepatitis E Virus (HEV), among which HBV, HCV, HDV are bloodborne viruses that are commonly transmitted through inadequate sterilization of medical equipment and injection devices and the transfusion of contaminated blood and unscreened blood, while HAV and HEV are transmitted via the fecal-oral route after consuming contaminated food and beverages [34,35]. The general symptoms of hepatitis viruses commonly include fever, fatigue, malaise, vomiting, nausea, abnormal pain, joint pain, and jaundice, but HAV, HBV, and HCV have been reported for their linkage to the development of coronary heart disease and atherosclerosis [35,36,37,38]. The clinical data clearly showed the increased incidence of coronary heart diseases and atherosclerosis after infection with HAV, HBV, or HCV, and the data implied that hepatitis seropositivity is involved in the development of heart disease [36,37,38]. A clinical study, which was conducted with 391 patients, showed a higher prevalence of coronary artery disease in HAV-seropositive patients compared to 74% of HAV-seronegative patients [36]. Similarly, the association between HBV antigen seropositivity and atherosclerosis has been observed [37]. In addition, it has been reported that carotid atherosclerosis was diagnosed more prevalently within the chronically HCV-infected patients [38]. Thus, it is certain that hepatitis virus infections are a risk factor for the occurrence of atherosclerosis and cardiac disease, but the detailed mechanisms of the enhanced pathogenesis induced by hepatitis viruses are not sufficiently defined. It is roughly understood that inflammation linked with the hyperactivation of cytokines, such as IL-1β, IL-6, IL-10, and TNF-α causes pathogenesis of coronary heart disease and atherosclerosis [39,40]. Specific treatment methods against each hepatitis virus do not exist yet. Instead, people can be vaccinated to become protected against HAV and HBV [41,42].

## 5. Herpes Simplex Virus (Human Herpesvirus 1 and 2)

Herpes simplex virus (HSV)-1 and -2 are members of the human Herpesviridae family and are known as human herpesvirus-1 and -2, respectively. It is estimated that HSV-1 has infected 3.7 billion people worldwide, and HSV-2 has infected 400 million people worldwide [43,44]. Autopsy, biopsy, metadata analysis, and laboratory research data confirmed that HSV infection can initiate and progress the development of atherosclerosis [45,46,47,48]. Higher detection of HSV-1 DNA in the atherosclerosis autopsies and biopsies implies that the virus is highly linked to the disease [45,46]. The meta-analysis also suggests that HSV can increase the incidence of atherosclerosis [47]. From human specimens, it was confirmed that HSV-1 was more significantly detected in atherosclerotic groups compared to non-atherosclerotic groups [48]. HSV upregulates lectin-like oxidized LDL receptor-1 (LOX-1), which is the major receptor protein of oxidized LDL (oxLDL), stimulates the uptake of oxLDL in endothelial cells, provokes lipid accumulation and its metabolism through the increased acquisition of saturated cholesteryl esters and triacylglycerols, induces the accumulation of coronary artery calcium, and causes the development of thrombosis, which are all related to the development of atherosclerosis [49,50,51,52]. It is certain that HSV is involved in the pathogenesis of atherosclerosis. Vaccines and specific treatments against those virus species are not yet available, but antiviral medications, such as acyclovir, famciclovir, and valacyclovir are the most popular medications to alleviate symptoms of people infected with HSV even though those medications cannot completely cure the infection [53,54].

## 6. Human Papillomavirus

Human papillomavirus (HPV) is known as the most important causal factor of cervical carcinomas, where 99% of cases are associated with HPV infection [55]. HPV can be categorized into two types: low-risk and high-risk, among which types 16 and 18 are the most oncogenic ones responsible for approximately 70% of cases [56,57]. In addition to cancer, it has been reported that HPV infection is also related to atheromatous arterial disease and cardiovascular disease; for example, a clinical study showed that 55% of 20 patients having atheromatous coronary arteries had HPV types 16 and 18, and the other study showed that 65% of 60 female patients diagnosed with coronary artery disease had HPV [58,59]. It is currently understood that overexpression of HPV E6 and E7 proteins can progress atherosclerosis by degrading p53 and inducing the proliferation of smooth muscle cells (SMC) in aortic tissues [60,61,62]. It has been studied that p53 plays a very important role in the development of atherosclerosis through its control of cell replication and proliferation mediating the development of the atherosclerotic lesion [62]. Vaccines against specific types of HPV are available, such as Cevarix^®^ targeting types 16 and 18, and Gardasil^®^ targeting types 6, 11, 16, 18, 31, 33, 45, 52, and 58 [63]. However, a universal vaccine working against all types of HPV has not been developed yet. Specific antivirals are also currently unavailable.

## 7. Human Cytomegalovirus (Human Herpesvirus 5)

Human cytomegalovirus (HCMV) belongs to the Herpesviridae family and is known as human herpesvirus-5 [64]. It is estimated that approximately 83% of the global population have been already exposed to the virus and had antibodies of IgG class in their serum [65]. Moreover, the fetus can also be infected, and HCMV is never fully cleared from the infected host and persists during the lifetime [66]. HCMV can be reactivated from latency and triggers various inflammatory stimuli, and cellular and physiological stresses [67,68].

HCMV has been linked to atherosclerosis and cardiovascular diseases, and clinical data confirmed the existence of HCMV in the arterial walls of patients having ischemic heart disease and its linkage to the development of coronary artery disease and atherosclerosis [69,70]. Even though the detailed mechanisms are unclear, it is true that HCMV proteins and genomes are found in atherosclerosis-associated vessels, and HCMV increases the incidence of atherosclerosis [71]. Atherosclerosis is provoked and aggravated by HCMV via CD36 expression to promote uptake of OxLDL, US28 expression to stimulate migration of SMC, which is highly involved in the pathogenesis of vascular diseases, the suppression of p53 to increase SMC proliferation, UL122 expression to provoke endothelial cell injury through the translocation of heat shock protein 60 (HSP60), and T-cell expansion and accumulation [71,72,73,74,75,76]. In addition, animal model studies confirmed that the transmission of cytomegalovirus can provoke and aggravate atherosclerosis by hyperactivation of mitogen-activated protein kinase (MAPK) pathways, increasing levels of IFN-γ and TNF-α, the upregulation of vascular cell adhesion protein 1, also known as vascular cell adhesion molecule 1 (VCAM-1), Intercellular Adhesion Molecule 1 (ICAM-1), and MCP-1, and the expansion of the lesion size [77,78,79]. No vaccine against HCMV is yet approved and licensed, and the specific treatments against the virus are not yet developed. Instead, antiviral medications, such as acyclovir, ganciclovir, valganciclovir, foscarnet, cidofovir, and letermovir are available to alleviate the symptoms of people infected with HCMV even though those medications cannot completely cure the infection [80,81,82].

## 8. Human Immunodeficiency Virus

Atherosclerosis is highly linked to human immunodeficiency virus (HIV) infection in that HIV-positive patients show a higher prevalence of atherosclerosis than the HIV-negative population [83]. Atherosclerosis can be developed after HIV infection mainly by the provoked inflammation and hyperactivation of cytokines inducing the recruitment of immune cells, ER stress, and apoptosis of foam cells. HIV infection can elevate interleukin-1 (IL-1), IL-6, interleukin-12 (IL-12), interleukin-18 (IL-18), IFN-γ, and MCP-1, which stimulates TNF-α and -β, and the nuclear factor kappa-light-chain-enhancer of activated B cells (NFκB) [84,85,86]. These inflammatory mediators contribute to the recruitment of various immune cells, such as monocytes differentiating into macrophages, which are responsible for lipid engulfment to promote transformation to atherosclerotic foam cells [86]. It is speculated that oxLDL, MCP-1 production, increased calcium levels, and ER stress in aortic endothelial cells cause apoptosis of the foam cells and plaques in the arteries [87,88,89]. Antivirals to treat HIV exist, but it has been reported that some drugs, such as ritonavir or efavirenz, can cause dyslipidemia and atherosclerosis [90,91]. Pre-exposure prophylaxis (PrEP) treatment was the only option to prevent HIV infection, but the U.S., the FDA has recently approved the first injection drug Apretude to prevent HIV infection [92,93,94,95,96]. Additionally, it has been reported that various antiretroviral drug treatments including Ritonavir, Saquinavir, Nelfinavir, Indinavir, Efavirenz, and Tenofovir alafenamide can increase the levels of human blood lipids and the incidence of atherosclerosis [97]. It is thought that those antiretroviral drugs induce oxidative stress, change lipid metabolism, increase proinflammatory cytokines, and express adhesion molecules, which can deteriorate endothelial function and aggravate atherosclerosis [98].

## 9. Discussion

Based on autopsy, biopsy, metadata analysis, animal study, and molecular biology research data, it is certain that specific viral infections are highly linked to the pathogenesis of atherosclerosis. Nevertheless, the detailed molecular mechanisms that initiate, trigger, develop, and aggravate atherosclerosis with viral infections are not completely understood yet. Some viruses, such as HSV-1, HSV-02, HCMV, and HCV show traits that imply their direct and indirect effects on the development of atherosclerosis, but the involvement of HPV and influenza viruses is still uncertain or undetermined [99]. Viruses can directly induce atherosclerosis via their direct interactions by infecting vascular cells, multiplying in the atherosclerotic plaque to accelerate the infection, while an indirect effect can occur in the non-vascular sites with increased levels of cytokines and immune proteins which lead to the development of atherosclerosis; thus, the viral genome can be isolated from the atherosclerotic plaques if the direct viral effects occur, but their genome cannot be isolated from the atherosclerotic plaques in the cases caused by indirect viral effects [99,100]. For the direct effects, the viral infection activates and accelerates the innate immune responses, and the activated innate immune cells can help to further express various pro-inflammatory and/or prothrombotic cytokines, such as IL-1β, IL-6, INF-γ, and TNF-α, which can activate and promote macrophages within the vascular cells; LDLs are also found in the form of oxLDL, and these oxLDLs in the infected cells and tissues are phagocytized by macrophages, which will induce the generation of the foam cells and plaque [99,100]. For the indirect effects, viral infection generally occurs in the non-vascular sites, and activates innate immune responses inducing the expression of pro-inflammatory and/or prothrombotic cytokines, such as IL-1β, IL-6, INF-γ, and TNF-α in the non-vascular site; macrophages circulate and move to vascular cells and become imported to the site to induce phagocytosis of oxLDLs and the formation of foam cells and plaque [99,100].

**Figure 1:**
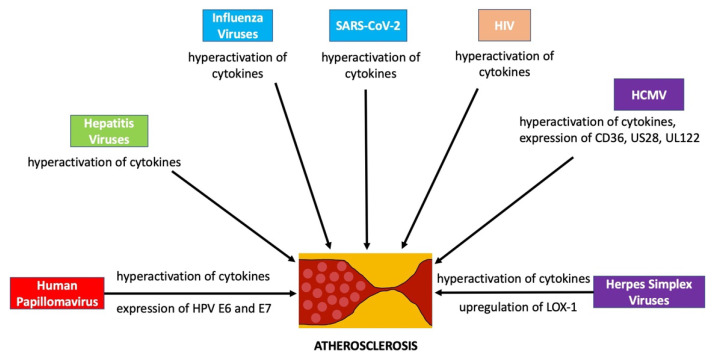
The Currently Defined Mechanisms of Atherosclerosis Development by virus infection. All viruses can induce the hyperactivation of cytokines and activate immune cells in a host, while some viruses exhibit additional unique mechanisms in the development of atherosclerosis. SARS CoV-2, severe acute respiratory syndrome coronavirus-2; HIV, human immunodeficiency virus; HCMV, human cytomegalovirus; HPV, Human Papilomavirus; LOX, lectin-like oxidized LDL receptor.

Although viruses can be detected in atherosclerotic sites for the direct effects or non-vascular sites for the indirect effects, which strongly suggests that viral infection is closely linked to the development of atherosclerosis, many aspects of their molecular effects are still presumably attributed to the inflammatory and immunological events which are usually observed in the infection sites, such as the hyperactivation of cytokines and inflammations in a host along with some extra features as summarized in Figure 1. High viral replications in the host can lead to the hyperactivation of proinflammatory cytokines, various immune components, and modulators. Such increased secretions of the chemokines and interleukins, overexpression of various immunogenic modulators and receptors, and hyperactivation of immune cells along with arterial stiffness and dysfunction and disruption of endothelial tissues can provoke and progress atherosclerosis after viral infections. Thus, it can be understood that the development atherosclerosis by viral infection is mainly triggered by the hyperactivation of cytokines with some additional unique mechanisms given by the specific viruses. Based on these mechanisms, it can also be understood why so many patients with COVID-19 showed serious symptoms of cardiovascular diseases and why many people being vaccinated with the COVID-19 vaccine booster shots have suffered from serious symptoms of cardiovascular disease right after vaccination. It is suggested that the exaggerated immune reactions caused by COVID-19 vaccine booster shots may provoke similar mechanisms and reactions as the viral infections linked to the development of atherosclerosis. Therefore, the need to further investigate the underlying mechanisms of the development of atherosclerosis by viral infections is truly notable, and safer vaccines to protect people and novel antivirals to completely cure the infections must be further investigated in the scope of the pathogenesis of heart disease, including atherosclerosis and should be ultimately developed to improve the health of people worldwide.

## 10. Conclusions

In this paper, we reviewed the specific viruses linked to the development of atherosclerosis with the specific purpose of preparing a condensed manuscript in a mini review format to help students, scientists, and healthcare professionals efficiently and rapidly learn about the viruses linked to atherosclerosis. Atherosclerosis is a chronic inflammatory disease, and it manifests with the thickening of artery walls and their narrowed channels through the accumulation of plaque [1,2]. It is widely known that atherosclerosis can be developed by smoking, a high cholesterol diet, hypertension, or hyperglycemia [4]; but viral infection can also trigger and aggravate atherosclerosis development [5,6,7]. However, information related to the underlying mechanisms in atherosclerosis development via viral infection is still very limited, and the current understanding mainly relies on statistical reports from various clinical studies. Many aspects of their molecular effects are presumably attributed to the general inflammatory and immunological events depicted in the Figure 1. For example, many patients with COVID-19 showed symptoms of cardiovascular diseases clinically, and it has already been proposed by other research groups that SARS CoV-2 is potentially linked to atherosclerosis and that the virus can be a risk factor for atherosclerosis development [8,9], but the underlying mechanisms are not completely defined [101,102,103]. Therefore, the underlying mechanisms in atherosclerosis development by viral infection should be further investigated and defined to obtain a more precise knowledge of the relationships between atherosclerosis and viral infection. In addition, safer vaccines to prevent viral infection and enhanced antivirals to cure viral diseases must be further studied and developed not only to protect people from viral infection but also to prevent the development of heart disease, including atherosclerosis, after viral infection.

## Data Availability

Not applicable.
